# Prevalence of neurogenetic disorders in the North of England

**DOI:** 10.1212/WNL.0000000000001995

**Published:** 2015-10-06

**Authors:** David Bargiela, Patrick Yu-Wai-Man, Michael Keogh, Rita Horvath, Patrick F. Chinnery

**Affiliations:** From the Institute of Genetic Medicine, Newcastle University, Newcastle upon Tyne, UK.

## Abstract

**Objective:**

Genetic disorders enter the differential diagnosis of common neurologic diseases, but their overall prevalence is not known. We set out to determine their minimum prevalence.

**Methods:**

Meta-analysis of epidemiologic data gathered from the same geographic region in the North of England.

**Results:**

Monogenic neurologic disorders affect at least 90.9/100,000 (95% confidence interval 87.6–94.3), or 1 in 1,100 of the population in Northern England.

**Conclusion:**

As a group, neurogenetic disorders are not rare. These findings have implications for clinical service delivery.

Recent advances in molecular diagnostics have expanded our understanding of the heritable basis of neurologic disease. The possibility of a highly penetrant mutation in a single gene enters the differential diagnosis of the most common neurologic disorders. If the inherited disorder presents late in life, or has a reduced penetrance, then there may be no affected relatives, so the absence of a family history does not preclude a genetic diagnosis. The widespread availability of inexpensive DNA tests has made it easier than ever to reach a molecular diagnosis in a patient with a suspected neurogenetic disorder, but the overall prevalence of these diseases has not been established. Herein, we present the results of a systematic review of neurogenetic disorders in the North of England, where the stable population has provided an ideal substrate for genetic epidemiology.^1^

## METHODS

We reviewed the reported prevalence of neurogenetic disorders, here defined as neurologic conditions known to be caused by highly penetrant mutations in a single gene (so-called “monogenic” or “single gene” disorders). We did not include data on common neurologic conditions, such as Alzheimer disease, Parkinson disease, motor neuron disease (amyotrophic lateral sclerosis [ALS]), or inherited dystonias, in which only a minority of cases are caused by a single highly penetrant gene defect, although their likely contribution is discussed later in the article.

A systematic literature review was undertaken by searching PubMed (MEDLINE) listings from January 1966 to April 2015 for relevant articles using the following terms: “ataxia,” “neuropathy,” “muscle disease,” “myopathy,” “dystrophy,” “Huntington,” “mitochondrial,” “optic atrophy,” or “hereditary spastic paraparesis” combined with the terms “epidemiology,” “prevalence,” or “incidence.” There was no language restriction. The final reference list was generated on the basis of relevance to the topics covered in this review. Priority was given to studies based on a molecular genetic diagnosis rather than those based on clinical or biochemical criteria alone. Articles containing disease prevalence statistics for the North of England (see the figure for region definition) were included. The minimum point prevalence for each disorder was calculated using live affected cases and UK Office for National Statistics' population estimates for the North of England (appendix e-1 on the *Neurology*® Web site at Neurology.org) for the precise census date in each study.^2^ Exact 95% confidence intervals (CIs) were calculated by the Clopper-Pearson method.

**Figure:**
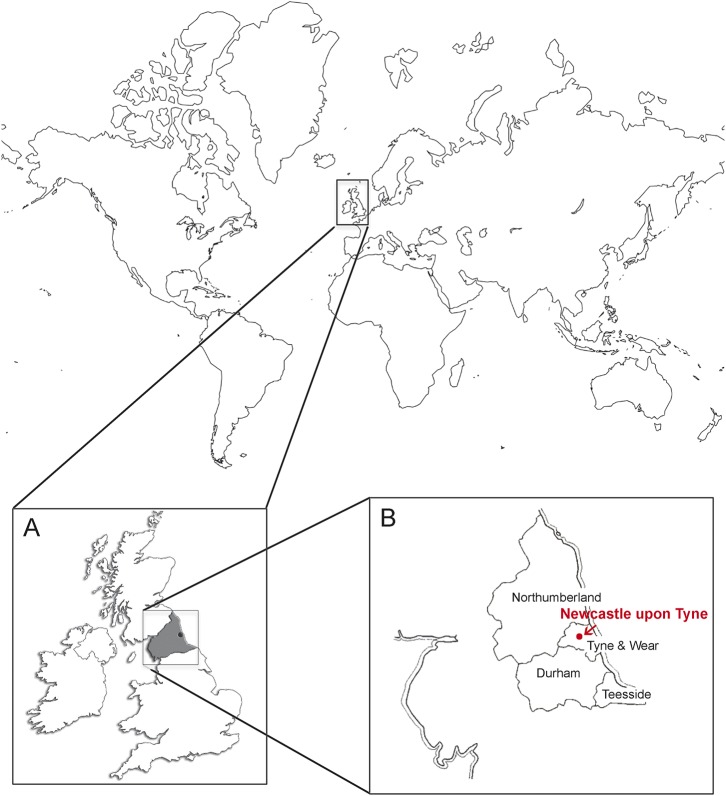
North East England Map of the world showing the United Kingdom (A) and the North of England (shaded in A). (B) North of England region and Newcastle upon Tyne and the population regions specified in appendix e-1.

## RESULTS

The systematic review identified the most up-to-date published prevalence data for the majority of neurogenetic disorders from the North of England between 1966 and 2015 (table). For hereditary spastic paraparesis (HSP), there were no published data from the United Kingdom and so we calculated a prevalence estimate from the Northern England neurogenetic database, an up-to-date census of patients with genetic conditions maintained by the Northern Genetics Service, which is based in Newcastle upon Tyne and serves the geographic region shown in the figure. Overall, the minimum point prevalence for neurogenetic disorders in the North of England was 90.9/100,000 (95% CI 87.6–94.3). The specific studies contributing to this result are described below.

**Table:**
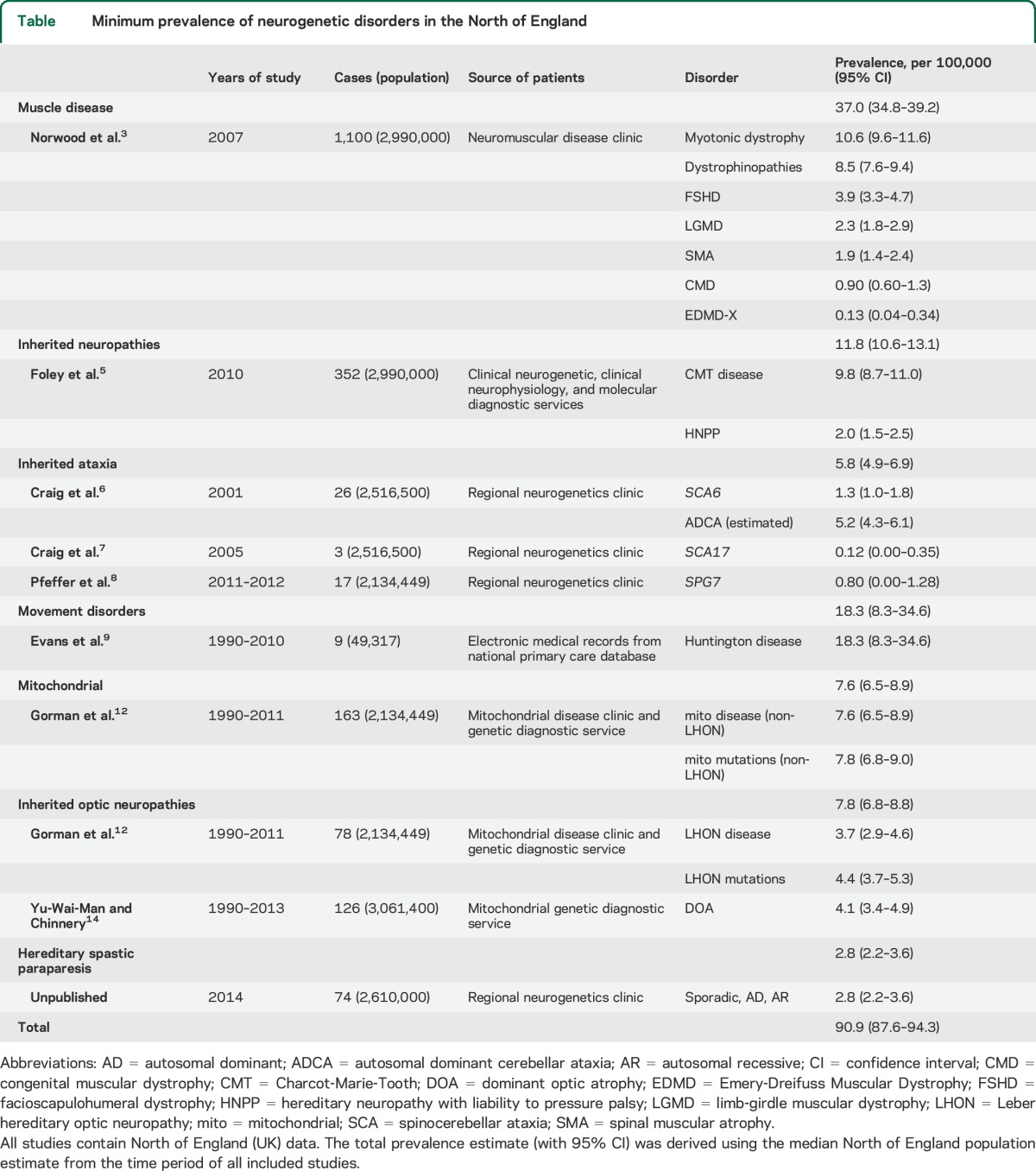
Minimum prevalence of neurogenetic disorders in the North of England

### Inherited muscle disease.

The point prevalence of all causes of genetic muscle disease was determined by Norwood et al.^3^ by a survey of a specialist muscle disease clinic in the North of England. From a clinic population of 1,100 patients registered with the Newcastle neuromuscular team, the combined point prevalence for all genetic muscle disease of 37.0/100,000 (95% CI 34.8–39.2) was established. The 5 main conditions represented in the population were myotonic dystrophy with a point prevalence of 10.6/100,000 (95% CI 9.6–11.6), dystrophinopathies at 8.5/100,000 (95% CI 7.6–9.4), facioscapulohumeral muscular dystrophy of 3.9/100,000 (95% CI 3.3–4.7), limb-girdle muscular dystrophy 2.3/100,000 (95% CI 1.8–2.9), and spinal muscular atrophy 1.9/100,000 (95% CI 1.4–2.4). Twelve further muscle disorders made up the remaining diagnoses including congenital muscular dystrophy with a point prevalence of 0.90/100,000 (95% CI 0.60–1.3) and 4 patients had X-linked Emery-Dreifuss muscular dystrophy giving a point prevalence of 0.13 (95% CI 0.04–0.34).^3^

The prevalence of *GNE* (glucosamine [UDP-N-acetyl]-2-epimerase/N-acetylmannosamine kinase) myopathy, a rare distal myopathy with inclusion bodies estimated under the category of “distal myopathies,”^3^ was recently assessed separately in a Northern England cohort.^4^ Cases were identified from referral records to the Newcastle MRC Neuromuscular GNE diagnostic service, also based in the Northern Genetic Service. Genetics testing became available in 2010, after which the center received 87 referrals—64% from England, 13% Scotland, 14% from Northern Ireland, and the remaining referrals from abroad. Twenty-six cases of GNE myopathy were identified, 7 of these from England (3 from Northern England, 4 from Southern England), 10 from Scotland, 8 from Northern Ireland, and 1 from the Republic of Ireland. Using these population estimates, a point prevalence of GNE myopathy in Northern England can be calculated as 1/million (95% CI 0.0–2.9) and for the UK as 0.4/million (95% CI 0.0–0.6).

### Inherited neuropathies.

The most recent UK epidemiologic study of inherited neuropathies was published in 2012.^5^ The authors calculated the point prevalence of Charcot-Marie-Tooth (CMT) disease and hereditary neuropathy with liability to pressure palsy (HNPP) in a Northern England cohort using 3 databases, identifying cases using clinical, molecular diagnostic, and electrophysiologic criteria. From these, 352 individuals from 275 families were identified with a clinical diagnosis of CMT disease or HNPP. A minimum prevalence of CMT disease in the Northern region was estimated at 9.8/100,000 (95% CI 8.7–11.0) and for HNPP at 2.0/100,000 (95% CI 1.5–2.5), giving a combined minimum prevalence of 11.8/100,000 (95% CI 10.6–13.1).

### Inherited ataxias.

Two studies in the North of England region calculated point prevalence of *SCA6* and *SCA17* mutations, which both cause autosomal dominant cerebellar ataxia (ADCA). The first of these evaluated the prevalence of *SCA6* mutations,^6^ which lead to an adult-onset progressive syndrome of ataxia, dysarthria, and nystagmus. Twenty-six cases heterozygous for the CAG repeats were identified from 16 genealogically distinct families. Using their data, a minimum prevalence of ADCA due to *SCA6* can be estimated as 1.3/100,000 (95% CI 1.0–1.8) and in adults aged 45 years or older as 3.2/100,000 (95% CI 2.1–4.3). Because *SCA6* mutations account for approximately 20% of ADCA in the region, the minimum prevalence of ADCA in the general population can be calculated as 5.2/100,000 (95% CI 4.3–6.1). The same group conducted a further study assessing the prevalence of SCA17,^7^ a dominantly inherited ataxia with extrapyramidal features and dementia presenting with a Huntington disease (HD)-like phenotype. Ninety families with suspected HD and 192 families with undiagnosed ataxia were included and screened for *SCA17* mutations. Two of the 192 patients with undiagnosed ataxia, but none of the HD-like patients, were found to have (CAG/CAA)_n_ expansions larger than the control range. Using these data, a minimum prevalence of SCA17 was estimated as 0.12/100,000 (95% CI 0.00–0.35). There were no UK studies specifically studying the prevalence of autosomal recessive ataxia, apart from one focused on *SPG7* in North East England,^8^ which reported a prevalence of 0.80/100,000 (95% CI 0.00–0.13).

### Movement disorders.

The prevalence of HD in a UK cohort was estimated using the UK's General Practice research database, the world's largest computerized database of primary care medical records.^9^ Patients older than 21 years with a diagnosis of HD were included (n = 1136), and prevalence rates were calculated annually between 1990 and 2010. The prevalence of HD increased considerably between with this period, with a prevalence of 5.4/100,000 (95% CI 3.8–7.5) in 1990 up to a prevalence of 12.3/100,000 (95% CI 11.2–13.5) in 2010. These increased rates over time were predominantly represented in patients older than 60 years, whereby a prevalence in the 60 to 69 age group of 12.6/100,000 (95% CI 9.1–17.1) in 1990 rose to 24.2/100,000 (95% CI 21.1–27.5) in 2010 and in the 70+ group a prevalence of 7.2/100,000 (95% CI 4.8–10.4) in 1990 rose to 15.6/100,000 (95% CI 13.2–18.3) in 2010. Results were further analyzed on a regional basis showing that the North East of England and Scotland had the highest prevalence rates (18.3/100,000: 95% CI 8.4–34.6 and 16.1/100,000: 95% CI 10.8–22.9, respectively) while the lowest rates were found in London (5.4/100,000: 95% CI 3.0–8.8).

### Mitochondrial disease.

The prevalence of mitochondrial disease was initially estimated in 2000,^10^ and subsequently revised,^11,12^ based on referrals to a national diagnostic service for rare mitochondrial diseases in Newcastle upon Tyne in the North of England. The minimum prevalence of mitochondrial disease was established through molecular genetics testing for mitochondrial DNA (mtDNA) point mutations and deletions, and nuclear-genetic causes of mitochondrial disease in adults (defined as older than 16 years; *SPG7* and *OPA1* are included in the ataxia and inherited optic neuropathy sections of this report, respectively).^12^ Both adults and children were included to establish those at risk of mitochondrial disease. By excluding mtDNA mutations that cause Leber hereditary optic neuropathy (LHON, considered separately in the section “Inherited optic neuropathies” below), the minimum prevalence of non-LHON mitochondrial disease mutations in the population was calculated as 7.8/100,000 (95% CI 6.8–9.0) and the minimum prevalence of non-LHON mitochondrial disease as 7.6/100,000 (95% CI 6.5–8.9).

### Inherited optic neuropathies.

The 2 principal inherited optic neuropathies are LHON and autosomal dominant optic atrophy (DOA).

The prevalence of LHON was established through the prospective mtDNA genetic testing of patients with unexplained visual failure or suspected LHON in the Northern Genetic Service over a 21-year period (January 1990 to May 2011).^12^ Within this population, the prevalence of LHON was calculated as 3.7/100,000 (95% CI 2.9–4.6), while the prevalence of mtDNA LHON mutations, representing those “at risk” within the population, was 4.4/100,000 (95% CI 3.7–5.3).

The same center previously established the prevalence of DOA using North of England neurogenetic and ophthalmology clinical databases.^13^ Seventy-six patients from 22 families were identified with a clinical diagnosis of DOA, and *OPA1* gene sequencing was performed on this group. Those found to be negative for this were further screened for *OPA1* rearrangements and *OPA3* mutations. The majority (14/22, 63.6%) of families with DOA carried *OPA1* mutations while *OPA1* rearrangements were only found in one family and no *OPA3* mutations were detected in the study cohort. The calculated point prevalence estimate from this data for clinically determined DOA was 2.9/100,000 (95% CI 2.3–3.5) with the point prevalence of molecularly confirmed *OPA1*-positive DOA as 2.1/100,000 (95% CI 1.6–2.7). A recent update from the same group included a further 8 *OPA1*-positive and 5 *OPA1*-negative families resulting in a revised point prevalence estimate for DOA of 4.1/100,000 (95% CI 3.7–4.5).^14^

### Hereditary spastic paraparesis.

We found no published UK epidemiologic data on the prevalence of HSP. We identified cases in the North of England using the Northern Genetics Service database to identify patients with suspected and genetically confirmed HSP. All patients had brain and spinal cord imaging to exclude a structural or inflammatory cause for the spastic paraparesis, and all had normal CSF examination. From a population of 2.61 million (mid-2013 estimate) in the North East region, 176 individual cases from 55 genealogically distinct families with clinical HSP were identified. Of this group, 74 cases (44.3%) had a confirmed genetic diagnosis giving a minimum point prevalence of confirmed HSP of 2.8/100,000 (95% CI 2.2–3.6). This did not include patients with *SPG7* who were identified through their ataxic presentation and were included in the ataxia section above.

## DISCUSSION

Here we show that approximately 1 in 1,100 of the general population is directly affected by a “single gene” neurologic disorder. This figure is likely to be an underestimate for several reasons. First, most of the studies we included had ascertained cases through hospital referrals, and not by systematically investigating a community-based cohort. This approach introduces a bias toward younger, more highly motivated individuals who seek specialist advice about a rare diagnosis. Older patients may be less inclined to pursue a precise diagnosis in a specialist center, given that there may be little hope of a cure. Many of the disorders described here have an age-related penetrance, so the underascertainment of older individuals is likely. Prospective community-based studies usually yield higher prevalence figures, but are particularly challenging for rare diseases because of the very large sample size required to reliably estimate the prevalence.^15^ Second, some of the studies did not incorporate comprehensive genetic testing, raising the possibility that some of the cases were misdiagnosed as a noninherited disorder because there was no family history, and missed altogether. Family history is not a reliable guide to monogenic disease because a positive family history depends on the degree of relative affected,^16^ and even high penetrance mutations frequently present without a family history.^17^ Finally, it is also important to note that here we describe the results of several different studies performed at different time points. Most, but not all, of the studies were performed in the same geographic region but had subtly different forms of case ascertainment and encompassed different age groups (table).

Could our results be an overestimate? This is unlikely because most of the studies we included were performed in the North of England, which has a well-defined outbred stable population of largely white European extraction.^1^ Given the limited consanguinity, it is unlikely that the prevalence estimates of recessive neurogenetic disorders are unusually high. When taken together, our findings are therefore likely to have broader relevance for other similar populations. When compared with prevalence data from studies worldwide, most of our estimates fit comfortably within previously delineated ranges: an earlier estimate of the prevalence of inherited muscle disease was 1 in 3,000 or 33.3/100,000,^18^ and a recent review has estimated the worldwide prevalence of HSP and ADCA as 0.1–9.6/100,000 and 0.7–5.6/100,000, respectively.^19^ Prevalence estimates for sporadic idiopathic cerebellar ataxia and autosomal recessive cerebellar ataxia (ARCA) were not obtainable for our region but can be estimated from a study in South Wales, UK, a region with a stable population and similar demographic.^20^ From a group of 76 cases with late-onset cerebellar ataxia identified from general practice, hospital and health authority databases, as well as through personal notification from consultant neurologists, 4 were classified as ARCA, giving a prevalence estimate of 0.53/100,000 (95% CI 0–1.38). We note, when comparing these data with the worldwide review,^19^ the prevalence estimate for ARCAs represents the lower end of a worldwide range spanning from 0.1 to 7.2/100,000. As noted by the authors of the Welsh study,^20^ this is likely attributable to methodologic limitations in case ascertainment as well as the challenge of classifying patients with an undetermined family history. It was further restricted in considering only “late-onset” cerebellar ataxia (symptom onset at age 18 years or older), which is likely to significantly underestimate the overall prevalence of ARCA. In a similar way, estimates of the prevalence of mitochondrial disorders^10,12^ did not include affected pediatric cases. In the age group younger than 16 years, the minimum prevalence of mitochondrial disorders has been estimate at 5.0/100,000 (95% CI 4.0–6.2),^21^ indicating that, for these conditions, our quoted figures provide an underestimate. Likewise, with the almost exponential increase in disease gene discovery, the number of “single gene” neurogenetic disorders is on the increase,^22^ further emphasizing that the figures we report here are likely to be a conservative underestimate.

We deliberately did not include genetic forms of common neurodegenerative diseases in our review because of the difficulty in diagnosing inherited forms of the disorder, which may be clinically indistinguishable from sporadic cases. However, using UK prevalence data for the principal neurodegenerative disorders Alzheimer disease (UK prevalence 1,100/100,000, of which ∼110/100,000 [∼1%] are monogenic^23^), Parkinson disease (UK prevalence 300/100,000, of which ∼30/100,000 [∼10%] are monogenic^24,25^), motor neuron disease (ALS, UK prevalence 6/100,000, of which ∼0.3/100,000 [∼5%] are monogenic^26^), and *DYT1* dystonia (prevalence ∼5/100,000^27^), the combined overall minimum prevalence of neurogenetic disorders increases to ∼234/100,000, or at least 1 in 423 of the UK population. This figure does not include sporadic neurodegenerative diseases with a complex genetic architecture involving many different genetic loci, such as *APOE*, which has a moderately strong influence on the risk of developing Alzheimer disease. It also cannot include genetic forms of neurodegenerative disease due to hitherto unknown genes. This is important to recognize, given the recent unexpected finding that hexanucleotide repeat expansions in *C9ORF72* cause a substantial proportion of cases of ALS and other neurodegenerative diseases.^28,29^ For these reasons, even the prevalence of 1 in 423 is likely to be an underestimate.

These findings have major implications. For example, the average UK primary care practice will have at least 16 patients with single gene neurogenetic disorders on their records^30^ and with an aging population, these figures are likely to increase. Moreover, a UK neurologist will have on average 80 patients with a neurogenetic disorder within their referral area.^31^ The majority of the disorders discussed here have no treatment and cause progressive disability, often over several decades. The effect on families and medical and social services will be substantial. Second, our estimates do not account for the impact of the genetic diagnoses on at-risk family members, who may request genetic counseling and prenatal and preimplantation diagnosis to prevent recurrence. Finally, with the mainstream introduction of exome and whole genome sequencing in clinical practice, the number of unexpected predictive tests will also increase, placing even greater strain on clinical service provision, and emphasizing the need to develop treatments that can slow down or prevent these disorders.

## Supplementary Material

Data Supplement

Accompanying Editorial

## GLOSSARY

ADCA: autosomal dominant cerebellar ataxia
ALS: amyotrophic lateral sclerosis
ARCA: autosomal recessive cerebellar ataxia
CI: confidence interval
CMT: Charcot-Marie-Tooth
DOA: dominant optic atrophy
HD: Huntington disease
HNPP: hereditary neuropathy with liability to pressure palsy
HSP: hereditary spastic paraparesis
LHON: Leber hereditary optic neuropathy
mtDNA: mitochondrial DNA
