# Supplementary material for: Prevalence of neurogenetic disorders in the North of England
Source: Neurology. 2015 Oct 6;85(14):1195–201. doi: 10.1212/WNL.0000000000001995 (PMC4607600; doi:10.1212/WNL.0000000000001995)
Supplement: Data Supplement [file supp_85_14_1195__index.html]

Data Supplement 

# Prevalence of neurogenetic disorders in the North of England

## Data Supplement

One appendix; PDF file.

**Neurology® data supplements are not copyedited before publication. Published editorials and translations have been copyedited.  
 © 2015 American Academy of Neurology.  
  
 Files in this Data Supplement:**

- Appendix e-1 - PDF file
