# Supplementary material for: Prevalence of neurogenetic disorders in the North of England
Source: Neurology. 2015 Oct 6;85(14):1195–201. doi: 10.1212/WNL.0000000000001995 (PMC4607600; doi:10.1212/WNL.0000000000001995)
Supplement: Data Supplement [file supp_WNL.0000000000001995_Appendix_e-1.pdf]

# Population in the north of england region

| **County,** Borough | **2001** | **2002** | **2003** | **2004** |
|---|---|---|---|---|
| **Northumberland** | 307363 | 308499 | 309448 | 310140 |
| **Tyne and Wear** | 1086816 | 1086546 | 1084723 | 1082774 |
| **Cumbria** | 487795 | 488681 | 491329 | 494918 |
| **County Durham** | 493678 | 493538 | 493931 | 495147 |
| Darlington | 97894 | 98474 | 98911 | 99343 |
| Hartlepool | 90152 | 89993 | 90134 | 90317 |
| Stockton-on-Tees | 183795 | 184940 | 185699 | 185687 |
| **North Yorkshire (part)** | | | | |
| Middlesbrough | 141233 | 140090 | 139050 | 138451 |
| Redcar and Cleveland | 139159 | 138520 | 138565 | 138643 |
| | | | | |
| TOTAL | 3027885 | 3029281 | 3031790 | 3035420 |

All data from Office for National Statistics. Population Estimates for UK, England and Wal

**Population, mid-year**

| 2005 | 2006 | 2007 | 2008 | 2009 | 2010 | 2011 |
|---|---|---|---|---|---|---|
| 310752 | 311375 | 312960 | 314057 | 314489 | 315463 | 316278 |
| 1085369 | 1086495 | 1089457 | 1091129 | 1094638 | 1100024 | 1104141 |
| 497040 | 498813 | 500779 | 500916 | 500786 | 500165 | 499817 |
| 497218 | 499348 | 502960 | 505617 | 507311 | 510628 | 512994 |
| 100287 | 101509 | 102632 | 103694 | 104355 | 105028 | 105584 |
| 90457 | 90781 | 90969 | 91379 | 91530 | 91773 | 92088 |
| 186350 | 187270 | 187937 | 189039 | 189978 | 190902 | 191824 |
| | | | | | | |
| 138517 | 138169 | 138190 | 137885 | 137273 | 137667 | 138368 |
| 138177 | 137621 | 136940 | 136512 | 135867 | 135383 | 135164 |
| | | | | | | |
| 3044167 | 3051381 | 3062824 | 3070228 | 3076227 | 3087033 | 3096258 |

les, Scotland and Northern Ireland. Access from: http://www.ons.gov.uk/ons/publications/all-rel

| 2012 | 2013 |
|---|---|
| 316116 | 315806 |
| 1108212 | 1113577 |
| 499104 | 498070 |
| 514348 | 515957 |
| 105248 | 105396 |
| 92238 | 92665 |
| 192406 | 193196 |
| | |
| 138744 | 138939 |
| 134998 | 134945 |
| | |
| 3101414 | 3108551 |

eases.html?definition=tcm%3A77-22371.
