# Supplementary material for: Prevalence of neurogenetic disorders in the North of England
Source: Neurology. 2015 Oct 6;85(14):1195–201. doi: 10.1212/WNL.0000000000001995 (PMC4607600; doi:10.1212/WNL.0000000000001995)
Supplement: Accompanying Editorial [file supp_85_14_1195_v2_index.html]

Accompanying Editorial 

# Prevalence of neurogenetic disorders in the North of England

## Accompanying Editorial

**Neurology® data supplements are not copyedited before publication. Published editorials and translations have been copyedited.  
 © 2015 American Academy of Neurology.  
  
 Files in this Data Supplement:**

- Accompanying Editorial - PDF file
