# Supplementary material for: Prevalence of neurogenetic disorders in the North of England
Source: Neurology. 2015 Oct 6;85(14):1195–201. doi: 10.1212/WNL.0000000000001995 (PMC4607600; doi:10.1212/WNL.0000000000001995)
Supplement: Accompanying Editorial [file supp_WNL.0000000000001995_1191.pdf]

EDITORIAL

[An] enumeration shall be made…

Bryan J. Traynor, MD, PhD
Corrado Angelini, MD

Correspondence to Dr. Traynor: traynorb@mail.nih.gov

Neurology® 2015;85:1191–1192

The last decade has revolutionized our understanding of the genetic defects that give rise to neurologic diseases. This genomic knowledge has provided us with remarkable insights into the cellular mechanisms underlying neuronal dysfunction; furthermore, the genetic dissection of heterogeneous neurologic disorders, such as ataxias and hereditary spastic paraparesis, has influenced clinical services and trials. Even in late-onset diseases, and in individuals without an obvious family history, the importance of genetics is increasingly recognized. There is a pressing need to collect neuroepidemiologic data for rare genetic disorders and to develop new techniques that facilitate this collection.

Nevertheless, neurogenetic diseases are individually quite rare, a perception that has knock-on effects for patient care and allocation of health care resources. In the absence of a vocal lobby, health care administrators tend to downweigh resources assigned to rare diseases in favor of conditions regarded as occurring more frequently in the community. Pharmaceutical companies are also less likely to pursue drug discovery for conditions without a sizable market. This effect is magnified in centrally managed, single-payer health care systems such as those that exist in the British Isles. This data-driven, numbers-orientated approach misses a crucial point that neurologists are painfully aware of, namely, that the health care burden of neurogenetics diseases, taken as a group, is substantial. Furthermore, the disability associated with neurologic diseases means that the financial outlay per case is often high and over a long time period.[1] To date, we have lacked coherent epidemiologic data to make this counterargument.

In this issue of *Neurology*®, Bargiela et al.[2] address this gap in our knowledge by estimating the cumulative incidence of neurogenetic diseases in the North of England. To do this, they stitched together existing epidemiologic data for 20 diverse conditions with known genetic etiology, including muscle diseases, hereditary neuropathies, inherited ataxias, movement disorders, mitochondrial diseases, and hereditary spastic paraparesis. Individually, their prevalence rates range from 0.12 per 100,000 persons to 18.3 per 100,000 persons, but their cumulative prevalence is an imposing 91 per 100,000.[2] Applying these estimates across Britain suggests that there are nearly 60,000 individuals with neurogenetics conditions in the population at any one time. This information strengthens arguments for a larger health care investment on behalf of this group of neurologic patients.

The authors deserve credit for their innovative "gumbo" approach. The more purist epidemiologists among us may point out that the individual studies used by Bargiela et al. had different catchment areas, collected data over different time periods, and had nonuniform modes of case ascertainment, and thus may not be strictly compatible with each other. The authors implicitly acknowledge these methodologic shortcomings by referring to their estimate as a "minimum prevalence" and recognize that the true prevalence of neurogenetic conditions may be higher than reported.[2] This is a reasonable assumption, especially when one considers that the major stumbling block in most epidemiologic studies of rare diseases is incomplete case ascertainment. Furthermore, a formal epidemiologic study of neurogenetic diseases would require considerable time and money, and, we strongly suspect, would yield much the same conclusions.

The scope of neurogenetic conditions will continue to grow, as the genomics revolution matures and the 100,000 Genomes Project in the United Kingdom[3] and President Obama's Precision Medicine Initiative[4] are completed. The overarching vision of these Manhattan-scale projects is to usher in an era of personalized medicine, perhaps with gene therapy tailored to the specific underlying defect of the individual patient. That is a laudable, long-term goal, but a necessary first step is that "[an] Enumeration shall be made."[5]

STUDY FUNDING

This work was supported in part by the Intramural Research Program of the National Institute on Aging, NIH.

DISCLOSURE

B.J. Traynor has a patent pending on the clinical testing and therapeutic intervention for the hexanucleotide repeat expansion of C9orf72. C. Angelini reports no disclosures relevant to the manuscript. Go to Neurology.org for full disclosures.

See page 1195

From the Neuromuscular Diseases Research Section (B.J.T.), Laboratory of Neurogenetics, NIA, NIH, Bethesda, MD; and Fondazione Ospedale San Camillo IRCCS (C.A.), Venice, Italy.

© 2015 American Academy of Neurology. Unauthorized reproduction of this article is prohibited.

REFERENCES

1. World Health Organization. Neurological Disorders: Public Health Challenges. Geneva: WHO Press; 2006.
2. Bargiela D, Yu-Wai-Man P, Keogh M, Horvath R, Chinnery PF. Prevalence of neurogenetic disorders in the North of England. Neurology 2015;85:1195–1201.
3. Siva N. UK gears up to decode 100,000 genomes from NHS patients. Lancet 2015;385: 103–104.
4. Collins FS, Varmus H. A new initiative on precision medicine. N Engl J Med 2015;372:793–795.
5. US Constitution. Art/Amend I, §2.

© 2015 American Academy of Neurology. Unauthorized reproduction of this article is prohibited.
